# Improved Rat Heart Preservation Using High-Pressure Gaseous Perfusion with Oxygen–Xenon Mixture

**DOI:** 10.3390/pathophysiology32040058

**Published:** 2025-10-31

**Authors:** Alexander Ponomarev, Daniil Kuznetsov, Elena Mukhlynina

**Affiliations:** 1 Laboratory of Molecular Biology, Immunophenotyping and Pathological Morphology, Regional Children’ Hospital, 620149 Ekaterinburg, Russia; 2 InPres LLC, 620133 Ekaterinburg, Russia; 3 Laboratory of Biologic Motility, Institute of Immunology and Physiology of the Ural Branch of the Russian Academy of Sciences, 620078 Ekaterinburg, Russia; 4 Laboratory of Morphology and Biochemistry, Institute of Immunology and Physiology of the Ural Branch of the Russian Academy of Sciences, 620078 Ekaterinburg, Russia

**Keywords:** transplantation, preservation, heart, persufflation, xenon, organ storage, high-pressure

## Abstract

Background: To address limitations in static cold storage (SCS) of donor hearts, we developed the High-Pressure Gaseous Perfusion without Fluidic Preservation Media (HIPPER) method, along with the necessary equipment for its application. Methods: 33 Wistar rat hearts were split into five groups: (Control) static cold storage (SCS) in HTK solution, (Exp) HIPPER using oxygen–xenon gas mixtures of varying ratios (“Gas-A”: 1/9, “Gas-B”: 9/1, and “Gas-C”: 1/1), and (Air) HIPPER using air. Hearts were preserved for six hours, followed by a one-hour Langendorff assessment. Results: Beating was restored in 4/10 Control hearts, 15/15 Exp hearts across all gas mixtures (*p* = 0.001 Control vs. Exp), and 6/8 Air hearts. Among resuscitated hearts, the mean heart rates (in bpm) were 131 ± 10 (Control), 164 ± 21 (Air), and 226 ± 13 (Exp) (*p* = 0.001 Control vs. Exp; *p* = 0.015 Exp vs. Air). The mean left ventricular pressures (in mmHg) were 31 ± 5 (Control), 45 ± 9 (Air), and 73 ± 7 (Exp) (*p* = 0.002 Control vs. Exp; *p* = 0.014 Exp vs. Air), with dP/dT max/min showing consistent trends (*p* < 0.006 Control vs. Exp and Air vs. Exp). Infarct size in Exp group was also significantly reduced, averaging 39.6 ± 6.6% (Control), 12.6 ± 3.3% (Air), and 6.3 ± 0.7% (Exp) of total myocardium area (*p* < 0.014 for Control vs. all). Conclusions: as evidenced by both quantitative and qualitative data, HIPPER consistently outperformed SCS following six hours of storage of rat heart regardless of the gas mixture, highlighting its potential as a more robust preservation method.

## 1. Introduction

The increasing global life expectancy, especially in developed countries, leads to new challenges in healthcare, particularly in the field of organ transplantation. As people age, the wear and tear on individual organs—such as the heart, liver, or kidneys—becomes a limiting factor, outpacing the body’s overall ability to sustain longevity. Consequently, organ failure is emerging as a critical limiting factor in extending healthy lifespans, which is reflected in the growing of organ transplant waiting lists, the rising number of transplant procedures, and the broadening criteria for organ donation, including the increased use of donors after circulatory death (DCD). While heart and kidney transplants are becoming routine procedures in specialized hospitals, limited viability of donor organs outside the body leads to underutilizing of the potential donor pool.

The heart, in particular, is highly intolerant to ex vivo storage. To prevent undesirable tissue degradation during preservation, various storage techniques have been developed. The standard and most widely used method, static cold storage (SCS), is significantly time-limited. Guidelines of the International Society for Heart and Lung Transplantation, as well as other expert recommendations, discourage SCS of more than four hours due to primary graft dysfunction (PGD) and poorer outcomes [1,2,3,4]. This restriction imposes severe logistical challenges, limiting the geographic range of potential donors and reducing organ accessibility for recipients.

**Figure 1:**
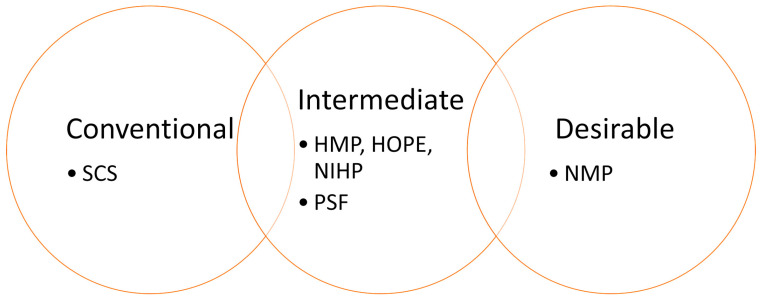
Existing heart preservation technologies. Modern heart preservation techniques are conceptualized as a spectrum, with simple Static Cold Storage (SCS) and complex Normothermic Machine Perfusion (NMP) at opposite ends. Intermediate methods, such as HMP, HOPE, NIHP, and PSF, occupy a middle ground, partially combining the advantages of both extremes.

One of the most promising approaches to increase ex vivo storage life of transplants is organ vitrification, which has demonstrated the ability to preserve a rat kidney for up to 100 days [5]. However, attempts to apply vitrification to heart preservation have limited success so far, with no reports of functional recovery and contractility [6,7]. Focusing on other heart preservation technologies, their range can be schematically represented as three intersecting circles (Figure 1). On one end of the spectrum is Static Cold Storage (SCS), a simple and robust technique based on a principle used for over 50 years: perfusing an organ with a cold preservation solution. Nevertheless, SCS is the least effective option for extended preservation, even though more than 167 different solution formulations have been created [8]. At the opposite end lies normothermic machine perfusion (NMP), a cutting-edge organ-preserving technique, which utilizes near-physiological perfusion with autologous blood at 35–37 °C [2,3,9]. However, NMP is expensive, technically complex, and requires highly skilled personnel [10,11]. Intermediate preservation techniques, including hypothermic machine perfusion (HMP) [12,13], non-ischemic heart preservation (NIHP) [14], and hypothermic oxygenated perfusion (HOPE) [1], share a common principle: the perfusion of complex solutions through the organ’s vasculature at low temperatures. They effectively balance procedural simplicity with enhanced preservation outcomes. These methods have demonstrated efficacy in large animal models, such as porcine hearts, and are currently under clinical investigation [1,14,15,16].

In this study, we report a novel heart preservation technique inspired by the concept of antegrade heart persufflation (aPSF) [15,16,17,18]. aPSF involves perfusion of coronary vessels by gaseous oxygen or gas mixtures (e.g., Carbogen) through aortic bulb. Early studies on porcine hearts showed that aPSF could preserve hearts for up to 14 h in both acute and chronic experiments [16,17]. However, despite promising results, aPSF has not gained clinical adoption in heart transplant preservation over the past two decades. Barriers to the adoption of aPSF for heart preservation may stem both from the stigmatization of the technology among clinicians due to concerns about myocardial gas embolism [19] as well as technical challenges, such as the need for precise aortic cannulation and sealing of the aortic valve, which requires a unique seal tailored to a specific heart. Furthermore, establishing flow of gas through the organ raises concerns not only about aseptic and transport issues but also about the overall bulkiness of the system [20]. On the other hand, relatively recent clinical trials have confirmed the success of PSF for human liver preservation, and its technical aspects for pancreas preservation have been incorporated into the consensus guidelines of the European Society for Organ Transplantation [21,22,23,24].

Our approach, termed HIPPER (High-Pressure Perfusion), overcomes key limitations of PSF by introducing several critical innovations: (a) a hermetically sealed pressurized chamber for organ placement, (b) a closed-loop gas perfusion system utilizing a pre-compressed gas mixture prevents open gas outflow, (c) a proprietary gas perfusion algorithm, and (d) a unique gas mixture containing xenon (Xe) and oxygen (O_2_). While the critical role of O_2_ is undeniable, the selection of an adjuvant gas remained unclear. We chose Xe due to its well-documented protective properties and established use in clinical practice [25,26]. A critical question remained: the optimal concentration of Xe to achieve therapeutic effects without toxicity. For instance, a recent study demonstrated that dysregulation of MitoKATP channels—whether through increased or decreased activity—leads to muscle degradation, underscoring the need for precise modulation [27]. This is particularly relevant given the known effects of Xe-mediated channel modulation [26]. Thus, different gaseous modalities—and even their ratios—may produce varying effects. For this reason, we employed O_2_-Xe mixtures at various ratios to identify potential differences.

In this comparative study, we used rat hearts as the experimental model and conventional SCS with HTK (Histidine–Tryptophane–Ketoglutarate) solution as the control. All experimental groups utilized the HIPPER technique with different gaseous mixtures: varying ratios of Xe:O_2_ and pure air. The inclusion of a pure air group was intended to isolate the effects of the gas mixture from those of the perfusion technique itself. For the initial validation of the HIPPER, we focused on a classical and interpretable design centered on the Langendorff method. This method allowed for quantitative assessments of the kinetics of reperfused organs and post-perfusion myocardial infarction [28].

## 2. Materials and Methods

### 2.1. Experimental Design

**Figure 2:**
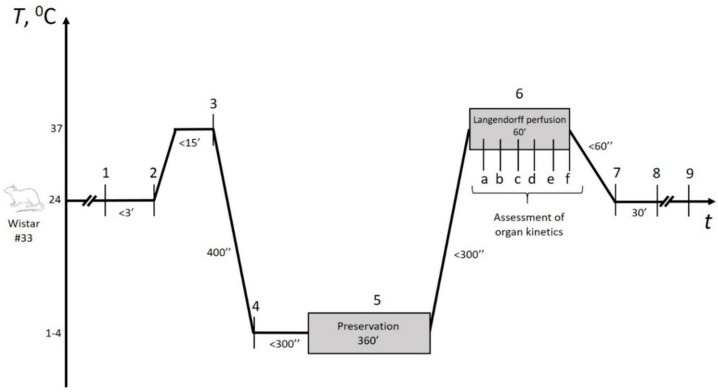
Experimental workflow. (1) Heart explantation, (2) Initial Langendorff perfusion for vasculature flushing and inclusion criteria assessment (refer to Table S3), (3) Cardioplegia induced by cold HTK solution perfusion, (4) Heart allocation into control (HTK solution maintained) and experimental groups; antegrade perfusion of gaseous mixtures under pressure restriction (91 ± 8 mmHg) with continuous monitoring, (5) Organ preservation phase, (6) Thawing procedure followed by Langendorff perfusion; hemodynamic assessments labeled A–F conducted every 10 min, (7) Organ sectioning into 1–2 mm slices and staining with Triphenyltetrazolium chloride (TTC), (8) TTC washout and infarct area examination using stereomicroscopy, (9) Data analysis. ’ denotes minutes. ” denotes seconds.

The experimental design is represented in Figure 2. Briefly, following cardioplegia within 5–7 min and heart relaxation, preservation began immediately, lasting no more than 5 min. In the control group, hearts were filled with HTK solution and immersed in iced saline to avoid contact-mediated ice injury, as recommended by cardiothoracic transplant guidelines [29]. In the experimental groups, the remaining 21-gauge aortic cannula was connected to the preservation device’s pump after the initial Langendorff perfusion, enabling continuous experimental gaseous perfusion throughout the preservation period. Details regarding the selection of gaseous mixtures and perfusion methods are provided in the following section. At the end of the storage period, control hearts were directly connected to the Langendorff setup (Radnoti, Dunedin, New Zealand), while experimental hearts underwent controlled depressurization first. The total preservation time was 360 ± 9 min, with no significant differences observed between the groups.

### 2.2. Animals and Ethical Considerations

The study protocol was approved by the ethical committee and the scientific board of the Institute of Immunology and Physiology of the Ural Branch of the Russian Academy of Sciences in Ekaterinburg, Russia. Wistar rats were bred and handled in accordance with the requirements of the European Convention for the Protection of Animals 2010/63/EU.

Given the proof-of-concept nature of this study and the absence of prior data, a preliminary power analysis was performed to estimate the required sample size. The calculation was based on the formula *N* = (*DF*/*k*) + 1, where *DF* (degrees of freedom) ranges from 10 to 20 and *k* is the number of groups. This formula, recommended as a rule of thumb in the absence of preliminary data [30,31], yielded a requirement of at least four animals per group using an average *DF* value of 15. Accordingly, a total of 33 male rats were randomly allocated into five experimental groups: (Control, *n* = 10) static cold storage (SCS) in HTK solution, (Exp) HIPPER using oxygen–xenon gas mixtures of varying ratios (“Gas-A”: 1/9, “Gas-B”: 9/1, and “Gas-C”: 1/1, *n* = 15), and (Air, *n* = 8) HIPPER using air) with baseline parameters summarized in Table S1. Statistical comparisons of key parameters showed no significant differences (Table S1).

### 2.3. Organ Retrieval

All retrievals began under deep anesthesia induced by barbiturate and ketamine followed by heparin administration. After confirming the absence of a pedal withdrawal reflex, a clamshell thoracotomy was performed, with a midline sternotomy extended to the costal margins and bilateral rib incisions along the anterior axillary lines. The anterior chest wall was retracted, and the pericardium was opened for vessel and organ access. The heart was excised by transecting the ascending aorta (below the brachiocephalic artery) and both venae cavae, then transferred to a dissection dish with iced Krebs–Henseleit buffer for hypothermic cardioplegia. Extraneous tissue was removed, and a 21-gauge blunt cannula was secured in the aorta, ensuring proper sealing without contact with the aortic valve leaflets. All manipulations were completed within 2 min on average to alleviate suffering if any. None of specific sacrificing methods were utilized due to predefined circulatory death under deep anesthesia. The heart was then mounted on the Langendorff rig and perfused with 37 °C Krebs–Henseleit buffer under constant pressure for a 10-min stabilization period (7th column in Table S1), during which inclusion criteria were assessed (Table S3). Perfusion was then switched to +4 °C HTK cardioplegic solution (Custodiol^®^, Dr. Franz Köhler Chemie GmbH, Bensheim, Germany) under constant flow for up to 5 min, maintaining pulsatile pressures of 60–80 mmHg.

### 2.4. HIPPER Technique–Gaseous Mixtures

The core concept of High-Pressure Perfusion (HIPPER) is to utilize a sealed chamber to house the organ under elevated pressure in a gaseous mixture. Furthermore, gas flow under high pressure is established through the vasculature, ensuring deep organ penetration. This requires sufficient, but not excessive, pressure to overcome vascular resistance and an optimized gas composition. We employed binary mixture of O_2_ and Xe considering the rationale discussed in introduction as well as our earlier results in RBC storage [25,32,33].

To explore the optimal Xe:O_2_ ratios, three compositions were tested: 9:1 (Gas A), 1:9 (Gas B), and 1:1 (Gas C). To verify the feasibility of these ratios, we propose the following equations, involving *P_pres_* (minimum chamber pressure in bar), Xe:O_2_ ratio, chamber volume, as well as organ-specific requirements for the preservation period.

(1) $$P_{pres}=\frac{{PV}_{O_{2}}}{F_{O_{2}}\cdot V_{c}}+1,$$

$F_{O_{2}}$ is O_2_ fraction in gaseous mixture,

$V_{c}$ is the inner volume of the device (mL),

${PV}_{O_{2}}$ is O_2_ demand by the organ within the expected preservation time (mL), which can be estimated as:

(2) $${PV}_{O_{2}}=\frac{m\cdot t\cdot {MV}_{O_{2}}}{A_{1}\cdot A_{2}^{(T_{1}-T_{2})/10}},$$

where m is the organ weight (g),

t is the expected preservation time (min),

${MV}_{O_{2}}$ is the organ’s O_2_ requirement under physiological conditions (mL/min/tissue weight in g), derived from literature,

$A_{1}$ is a coefficient, reflecting a decrease in O_2_ demand in the relaxed condition (without contraction) at physiologic temperature (e.g., 2.0–4.0 for rat’s heart). $A_{1}$ = 1, for organs with no contraction.

*A*_2_ is a coefficient, reflecting a decrease in O_2_ demand when the organ is placed in a hypothermic condition. Estimation is based on Q_10_ rule [34], yielding 2.0–3.0,

$T_{1}$ is the physiological organ temperature (°C), and

$T_{2}$ is the preservation temperature (°C).

The estimations show that for device design (200 mL of useful volume) the O_2_ content in all the mixtures was sufficient without the need to achieve extreme pressures, even for Gas A. In all scenarios, the estimated pressures did not exceed three bar, which was determined non-harmful in preliminary tests.

As a final step, we estimate the required frequency of gas exchange *N* within the organ’s vasculature in relation to pump performance:

(3) $$N=\frac{{PV}_{O_{2}}}{P_{pump}\cdot t},$$

where $P_{pump}$ is the pump performance (mL/s),

t is the expected preservation time (min),

${PV}_{O_{2}}$ is O_2_ demand of the organ within the expected preservation time (mL)

*N* is the frequency of the pump’s operation, measured in seconds of pump operation per minute time, and depending on device specifications. The ideal pump performance should align with both organ anatomy and O_2_ demand. For example, since the calculated amount of O_2_ required for the entire six-hour preservation of a rat heart does not exceed 100 mL, a pump performance of 1 mL/s is sufficient to switch it on once every 3.6 min for 1 s. In the present study, the pump was switched on for 1 s every 2 min.

### 2.5. Device Description

**Figure 3:**
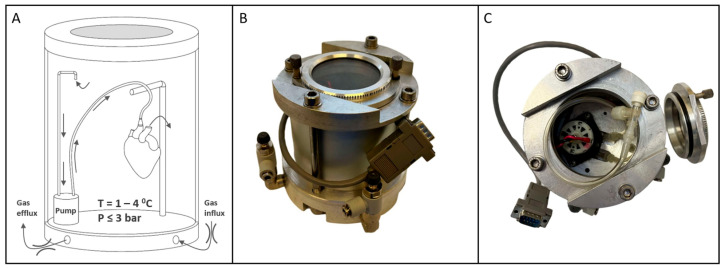
Custom chamber for HIPPER organ storage. (**A**) Schematics of the device and its operation. (**B**) Isometric frontal view of the device. (**C**) Top view of the opened device showing the inner chamber with the pump, tubing, and organ connector. Paired arc lines on panel A represent throttles.

The device used in this study (Figure 3) featured an inner chamber for organ accommodation, equipped with a pump, perfusion components (tubes, connectors, suspension), and a glass window for observation during pressurization and depressurization. The window also allowed monitoring of cardioplegia solution exiting the vasculature at the onset of gaseous perfusion, ensuring proper closure of the aortic valve leaflets.

A thermoelectric cooler provided precise, continuous refrigeration, eliminating thermal fluctuations typical of pump-based systems and maintaining a stable temperature curve that mimics SCS conditions (in Figure S1). Temperature and pressure sensors connected to an external controller managed thermobaric conditions within the chamber and organ. Custom software (ver. 1.0) visualized real-time data, plots graphs, and ensures adherence to the storage protocol (in Figure S1).

### 2.6. Perfusion Mode—In Operando Organ Assessment Criteria

Following heart suspension on the Langendorff rig, perfusion began at constant pressure (100 mmHg) with carbogenized Krebs–Henseleit buffer (O_2_:CO_2_ = 9.5:0.5) at 37 °C. Hemodynamic parameters, including heart rate, coronary flow rate, and left ventricular isovolumetric pressure, were monitored every 10 min over a 60-min reperfusion period using sensors (AD Instruments, Dunedin, New Zealand) and recorded in LabChart ver. 8.1 (AD Instruments, Dunedin, New Zealand). A polyethylene balloon, inserted into the left ventricle via the left atrium and inflated to 5–10 mmHg end-diastolic pressure, mimicked isovolumetric conditions while minimizing myocardial damage. Pressure derivatives (dp/dt max/min) served as surrogate metrics for myocardial contraction and relaxation.

### 2.7. Post-Reperfusion Organ Assessment

Ischemia–reperfusion injury (IRI) was assessed after reperfusion by slicing hearts into 1–2 mm sections, staining with 1% 2,3,5-triphenyl-tetrazolium chloride (TTC) in saline for 30 min, and fixing with 10% formaldehyde. Infarct areas were visualized using a stereomicroscope (Bresser Advance ICD, Rhede, Germany) equipped with a camera (Levenhuk M800 plus, Tampa, FL, USA) and quantified via planimetry software (LevenhukLite, v. 3.7, Tampa, FL, USA). Infarct size was expressed as the ratio of light (infarcted) to total areas. Hearts without rhythm after preservation were excluded from IRI analysis.

### 2.8. Data Analysis

Proprietary software LabChart ver. 8.1 (ADInstruments, Dunedin, New Zealand) was used to collect data from sensors integrated into the Langendorff rig. Raw data were exported from LabChart as .txt files and analyzed using open-sourced R statistical software ver. 4.3.1 (Vienna, Austria) [35]. Data analysis was harmonized across all observations using an identical approach for data processing (R script can be provided upon request). Briefly, heart rate, coronary flow, pressure and its derivatives were calculated by determining local parabola maxima followed by estimation of moving averages (within ±5 s intervals) where applicable. Integral P(t) was approximated as the area under the entire 60-min data (area under the curve, AUC).

Descriptive statistics included mean ± SE for normal distributions and medians (Q1, Q3) for non-normal data. Dichotomous data were analyzed by Fisher’s exact test while continuous data were analyzed using ANOVA. Welch’s or Student’s (fed by common SD) *t*-tests were used for post hoc pairwise comparisons, with variance homogeneity assessed by Bartlett’s test and data distribution by Shapiro–Wilk test. All multiple pairwise comparisons were adjusted using Holm’s correction based on the number of comparisons. All tests were performed between subjects using two-tailed hypotheses. Cohen’s d-values were calculated as the difference between group means divided by the pooled standard deviation. Principal component analysis (PCA) was used to visualize and measure distances between groups in a 2D plot, and the resulting distance matrix was analyzed using permutational ANOVA (PerMANOVA) to derive *p*-values. Results were presented as boxplots or barplots, depending on data distribution. A significance level of *p* < 0.05 was used throughout.

Statistical power estimation was performed either by generating random samples with predefined means and SD followed by Student’s *t*-test, or using tests of proportions for binomial distribution for continuous and dichotomous data, respectively. To facilitate independent data analysis, we deposited all raw data in an external repository BioStudies (accession number S-BSST2120). The deposited archive includes detailed descriptions for navigating the raw data files as well as .txt files for two independent experiments.

## 3. Results

### 3.1. Preliminary Organ Assessment

**Figure 4:**
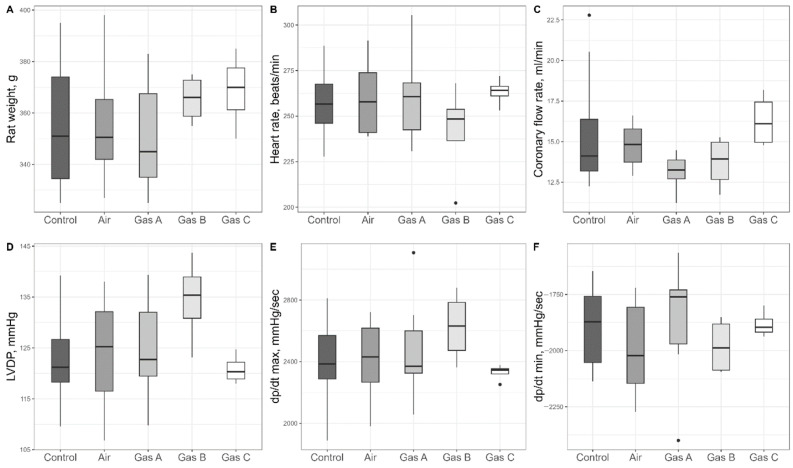
Assessment of inclusion criteria during the stabilization period, prior to preservation. (**A**) Animal’s weight in grams. (**B**) Heart rate in beats/min. (**C**) Coronary flow rate in ml/min. (**D**) Left ventricular developed pressure in mmHg. (**E**) Maximum derivative pressure of time (dP/dT max) in mmHg/sec. (**F**) Minimum derivative pressure of time (dP/dT min) in mmHg/s. Sample size: n = 10 (Control), n = 8 (Air), n = 7 (Gas A), n = 4 (Gas B), n = 4 (Gas C). Pairwise comparisons were made across all groups with no significant difference (*p* > 0.05).

Within the stabilization period (marked as 2 in Figure 2), we assessed initial parameters of the organs included in the study (Table S1 with selected parameters plotted in Figure 4); more than a dozen parameters were statistically compared, with no distinctions (*p* > 0.05).

### 3.2. Number of Hearts Successfully Resuscitated

**Figure 5:**
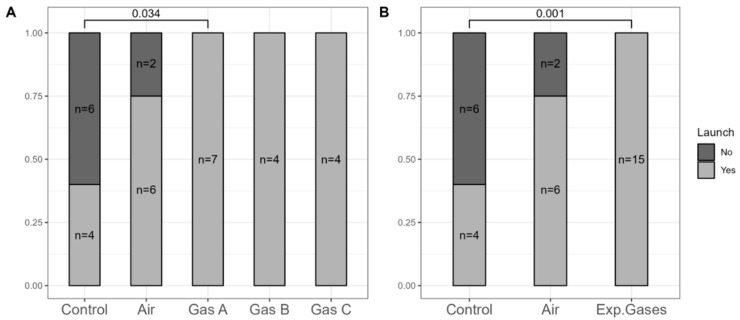
Number of hearts successfully launched post-preservation. (**A**) Measurements where all gaseous groups were separated. (**B**) Ad hoc comparison involving a single experimental gaseous group, reflecting pooled data of all gaseous groups. Square brackets denote statistically significant differences (*p* < 0.05).

To address the presence of non-beating hearts after reperfusion, we analyzed the resuscitation ratios post-preservation. Figure 5 shows the separation and aggregation of the experimental groups (Gases A, B, and C). For a more robust analysis, we combined all experimental gases into a single (pooled) group, reducing the *p*-value further—a reasonable step given that they only differed by Xe:O_2_ ratios.

All 15 hearts in the experimental groups were successfully resuscitated, compared to only 4 of 10 in the Control group and 6 of 8 in the Air group. Significant differences were noted between the Control group and Gas A (*p* = 0.034), with even greater significance (*p* = 0.001) when using the combined experimental gases variable.

### 3.3. Assessment of Organ Kinetics

**Figure 6:**
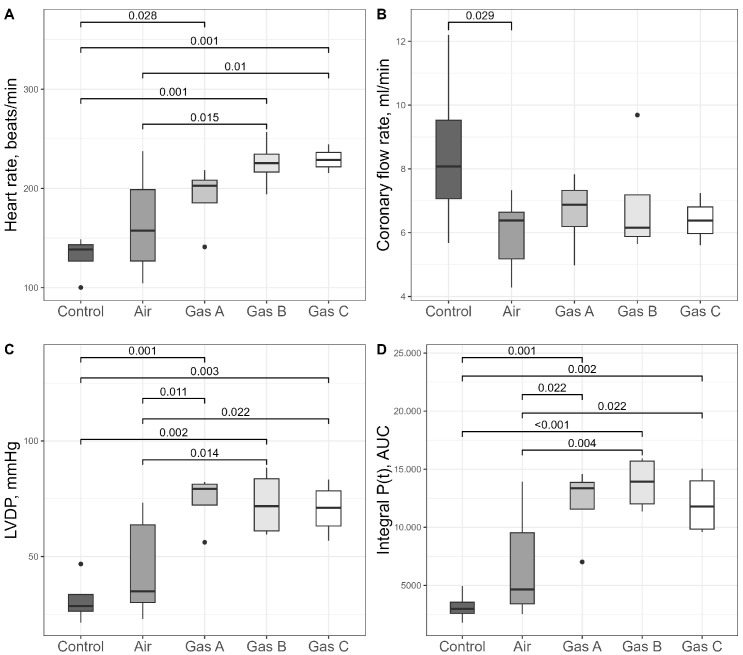
Assessment of heart viability post-preservation through hemodynamic integrity metrics. (**A**) Heart rate in beats/min. (**B**) Coronary flow rate in ml/min. (**C**) Left ventricular developed pressure in mmHg. (**D**) Integral P(t) represented by the area under the curve as indicated by sum of multiplied pressure over time. Square brackets denote statistically significant differences (*p* < 0.05). Sample size: *n* = 4 (Control, Gas A, Gas B, Gas C), *n* = 6 (Air).

We selected heart rate (HR), coronary flow rate (CFR), and the isovolumetric pressure developed by the left ventricle (LVDP) as direct measures of organ viability that reflect hemodynamics (Figure 6A–C). Significant differences were predominantly observed in HR (ANOVA, df1 = 4, df2 = 17, F = 5.82, *p* = 0.004) and LVDP (ANOVA, df1 = 4, df2 = 17, F = 6.37, *p* = 0.003) across the groups. We suggest that reporting Cohen’s d values allows readers to better estimate whether the differences between groups are biologically impactful. Therefore, we report these effect sizes alongside *p*-values. Following conventional guidelines, values greater than 0.5 are considered to denote a large effect size.

Gas C recorded the highest HR (229 ± 6 bpm), followed by Gas B (226 ± 13 bpm), with Gas A showing a decline (191 ± 17 bpm), though differences among experimental gases were not significant (*p* > 0.05). HR for the Control and Air groups were 131 ± 11 bpm and 164 ± 21 bpm, respectively, with significant differences noted between Air and Gas B (Cohen’s d = 1.39, *p* = 0.015), Air and Gas C (Cohen’s d = 1.56, *p* = 0.01), and Control vs. all experimental gases (Cohen’s d > 2.1, *p* < 0.03). The largest differences were observed for Gas B vs. Control (Cohen’s d = 3.97, *p* = 0.001) and Gas C vs. Control (Cohen’s d = 5.56, *p* = 0.001).

LVDP comparisons showed a similar trend, with an additional significant difference between Air and Gas A (Cohen’s d = 1.55, *p* = 0.011). LVDP values were 31 ± 5 mmHg, 45 ± 9 mmHg, 74 ± 6 mmHg, 73 ± 7 mmHg, and 71 ± 6 mmHg for Control, Air, Gas A, Gas B, and Gas C, respectively. Pairwise comparisons revealed difference among Control vs. all Gases (Cohen’s d > 3.2, *p* < 0.003) and Air vs. all Gases (Cohen’s d > 1.4, *p* < 0.02)

CFR (Figure 6B) showed no significance (ANOVA, df1 = 4, df2 = 17, F = 1.52, *p* = 0.24) but one significant difference on post hoc pairwise comparison: Control vs. Air (8.51 ± 1.37 mL/min vs. 5.98 ± 0.48 mL/min, Cohen’s d = 1.3, *p* = 0.029). Experimental gases had intermediate CFR values—6.64 ± 0.61 mL/min, 6.91 ± 0.94 mL/min, and 6.4 ± 0.35 mL/min for Gases A, B, and C, respectively—higher than Air but lower than Control.

The P(t) integral, a measure of overall kinetics that combines developed pressure and heart rate, was used to assess cardiac performance during reperfusion (Figure 6D). The P(t) integral mirrored the patterns observed for LVDP. One-way ANOVA revealed a significant overall effect (df1 = 4, df2 = 17, F = 7.53, *p* = 0.001). The values were as follows: Control (3168 ± 657), Air (6652 ± 1912), Gas A (12,077 ± 1715), Gas B (13,787 ± 1165), and Gas C (12,056 ± 1357). Cohen’s d values also highlighted significant differences. They were 3.4 (Control vs. Gas A, *p* = 0.001), 5.6 (Control vs. Gas B, *p* < 0.001), 4.2 (Control vs. Gas C, *p* = 0.002), 1.3 (Air vs. Gas A, *p* = 0.02), 1.8 (Air vs. Gas B, *p* = 0.004), 1.3 (Air vs. Gas C, *p* = 0.02).

### 3.4. Infarct Area

**Figure 7:**
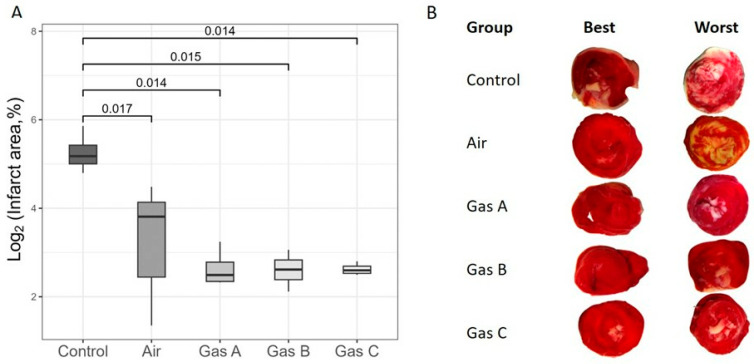
Comparative analysis of infarcted areas. (**A**) Planimetric estimation of infarct area across groups. Raw values expressed as percentages were log2-transformed for better visualization. (**B**) Transverse myocardial slices illustrating relative comparisons, determined by the best and worst slice match. Infarcted areas are represented by white/ivory color. Square brackets highlight statistically significant differences (*p* < 0.05). Sample size: n = 4 (Control, Gas A, Gas B, Gas C), n = 6 (Air).

To assess IRI, we calculated the infarct size of organs immediately following a 1-h reperfusion period (Figure 7). Due to a substantial *y*-axis disparity between the Control and Experimental groups, a log2 transformation was applied for visual clarity (Figure 7A), although we focused on the untransformed data in the text.

Infarct size estimations aligned with the organ kinetics trends, revealing significant differences between the Control and all other groups, including Air (Cohen’s d > 2.6, *p* ≤ 0.017 for all comparisons). So, Control vs. Air pair showed *p* = 0.017 (Welch, t = −3.69, df = 4.49, Cohen’s d = 2.6), Control vs. Gas A detected *p* = 0.014 (Welch, t = 4.99, df = 3.15, Cohen’s d = 3.5), Control vs. Gas B revealed *p* = 0.015 (Welch, t = 5.04, df = 3.1, Cohen’s d = 3.6) and Control vs. Gas C pair displayed *p* = 0.014 (Welch, t = 5.09, df = 3.01, Cohen’s d = 3.6). However, no significant differences were observed among Air and the experimental gases. Raw percentage values were 12.6 ± 3.3% (Air), 6.4 ± 1.1% (Gas A), 6.3 ± 0.9% (Gas B), and 6.2 ± 0.3% (Gas C), while Control values were markedly higher at 39.6 ± 6.6%, supporting the derived *p*-values and d-values.

Each tissue slice was analyzed to highlight qualitative differences and identify the best and worst outcomes. Figure 7B depicts transverse sections, arranged dichotomously and in groups. As expected, the worst cases were observed in the Control group, with infarct areas clearly distinguishable from all experimental groups, including Air due to the clearly visible ivory margins of varied thickness on all revived and 1-h reperfused hearts. It is worth noting the special attention required for the visible white areas representing the sites of papillary muscles on all the worst slices across experimental groups. This effect may be an artifact of the Langendorff technique, as the placement of a polyethylene balloon within the left ventricle can induce myocardial damage.

### 3.5. Surrogate Measures and Parallel Evaluation of Batched Variables

Surrogate measures of organ kinetics dP/dt max and dP/dt min (mmHg/s), followed the overall trends observed in other functional parameters. Statistical significance (*p* < 0.05) for these metrics aligned with earlier findings.

**Figure 8:**
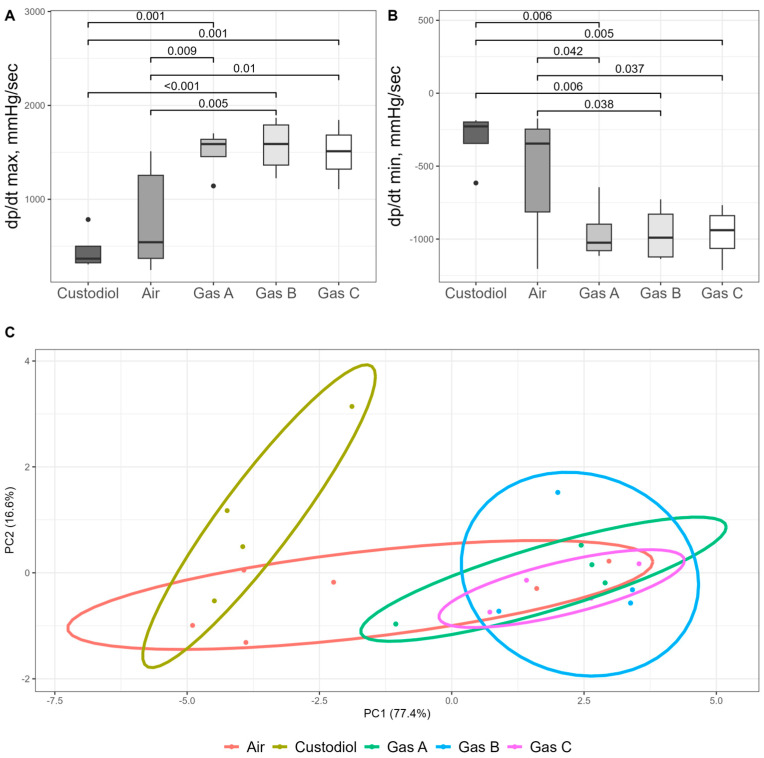
Surrogate measurements and batched evaluation of organ kinetics. (**A**) Maximum derivative pressure of time (dP/dT max) in mmHg/sec reflects contractility. (**B**) Minimum derivative pressure of time (dP/dT min) in mmHg/sec reflects relaxation. (**C**) PCA across groups fed by seven quantitative measures. Variables include HR, CFR, LVDP, derivatives, AUC, and infarct size. The analysis is represented by both PC1 and PC2 components, which collectively explain more than 90% of the total variance. All experimental gases nested in one cluster, while the Control group clustered at the opposite end, with the Air group positioned between them. Colors: Control—Yellow, Air—Red, Gas A—Green, Gas B—Blue, Gas C—Purple. Sample size: *n* = 4 (Control, Gas A, Gas B, Gas C), *n* = 6 (Air).

For dP/dt max, a measure of the heart’s contractile ability (Figure 8A), one-way ANOVA revealed a significant main effect (df1 = 4, df2 = 17, F = 7.51, *p* = 0.001). Post hoc analysis showed that values increased substantially across the experimental groups compared to the Control (456 ± 111), in the following order: Air (775 ± 231), Gas C (1494 ± 159), Gas A (1505 ± 125), and Gas B (1567 ± 151). All experimental gases demonstrated differences with both Control (Cohen’s d > 3.8, *p* < 0.001) and Air (Cohen’s d > 1.5, *p* < 0.01). Conversely, dP/dt min, which reflects the heart’s ability to relax, also showed a significant overall difference (ANOVA, df1 = 4, df2 = 17, F = 4.61, *p* = 0.01) but followed an inverse trend (Figure 8B). Exact values were −314 ± 102 (Control), −542 ± 175 (Air), −952 ± 106 (Gas A), −961 ± 100 (Gas B), −964 ± 97 (Gas C). Significant difference followed similar trend: all experimental gases vs. Control (Cohen’s d > 3.1, *p* < 0.006) and vs. Air (Cohen’s d > 1.1, *p* < 0.04).

One of our objectives was to identify the optimal gaseous mixture (A, B, or C) by evaluating all parameters in a blind, unbiased manner. To this end, Principal Component Analysis (PCA) was conducted (Figure 8C) using normalized values for HR, CFR, LVDP, derivatives, AUC, and infarct size.

The PCA plot represents data on a two-dimensional scale, with the X and Y axes accounting for over 90% of the variance. The plot reveals three distinct clusters: the Control group on the far left, partially overlapped by the Air group, which, in turn, intersects with the experimental groups (Gases A, B, and C). The experimental groups form aggregated but visually distinct clusters. Euclidean distances were calculated and compared across all observations, yielding significant differences between the Control and experimental groups (*p* < 0.04 for all comparisons). The Air group, as expected, showed no significant differences when compared with either the Control or experimental groups, reflecting partial cluster overlaps. Detailed PerMANOVA output with summary of test statistics is shown in Table S2.

## 4. Discussion

Previous research on gaseous organ perfusion, or PSF, has focused primarily on the liver, pancreas, and heart, establishing its utility in organ reconditioning and storage [15,18,21,22,24,36]. These methods typically involve perfusing organ vasculature with a gaseous mixture (or pure O_2_) under a pressure differential, while the organ is immersed in saline or preservation media. In this study, we demonstrated a novel HIPPER approach, which uniquely employs elevated gaseous pressure within a sealed chamber as the sole driver of a closed PSF circuit. This method excludes fluidic preservation media and is integrated into a semi-automated rig.

The core hypothesis of the HIPPER method is that the oxygen-rich microenvironment in a capillary creates a steep O_2_ gradient in the surrounding myocardium, which facilitates efficient oxygen diffusion into cells, thereby preventing the shift to inefficient, acidifying anaerobic metabolism. Calculations of O_2_ molarity in an 90 fL capillary volume (capable of holding one red blood cell), based on known data [37,38], demonstrate that even at atmospheric pressure, the number of O_2_ molecules in gas is almost 10 and 100 times higher compared to blood and to water (note, that SCS is based on aqueous preservation solutions), respectively. Considering that gas diffusion rates are four orders of magnitude higher than in liquids [38], the difference in gas exchange efficiency between gas and liquid becomes enormous.

Likewise, of crucial importance was the need to consider adjuvant gases because of the ambiguous role of pure oxygen as a sole fuel for HIPPER. To date, numerous gaseous modalities are known which modulate multiple steps within cell signaling cascades. These can be divided into gasotransmitters (including nitric oxide, hydrogen sulfide, sulfur dioxide, and carbon monoxide) and noble gases (such as argon, helium, and xenon (Xe)) [26]. We chose Xe as an adjuvant gas because it exerts modulation at various biological levels. For example, Xe has demonstrated organ and tissue protection, including greater recovery of left ventricular systolic function when combined with hypothermia [39]. Likewise, Xe acts on molecular levels; PKC-Ɛ serves as a key mediator of xenon-induced cardioprotection [40], and further downstream, Xe regulates p38 MAPK and MAPKAPK-2, linking its preconditioning properties to the regulation of the cardiac cytoskeleton and actin stress fibers via HSP27 [41]. Our preliminary experiments using Xe:O_2_ mixture demonstrated promising results. Hearts preserved under high-pressure gaseous perfusion remained viable and exhibited sustained beating after heterotopic transplantation to the abdominal aorta, unlike control, where no activity was observed even after electrical stimuli [File S1].

Based on these findings, a rigorous experimental plan was developed prioritizing well-defined control parameters and clinical relevance and minimizing confounding variables. To ensure robust analysis, we included an Air group to serve as a “semi-control,” enabling differentiation of effects from supplemental gases like Xe or its synergetic interaction with O_2_. We also standardized measurements using the Langendorff technique. One of the undeniable advantages of this technique is the decades of experimentation it provides. This includes the harmonization of individual elements such as left ventricular ballooning, the use of standard buffers (e.g., Krebs–Henseleit solution), and the pre-experiment stabilization phase for the heart. The clear benefit of this standardization is the strict control of experimental conditions, minimizing potential confounders that could impact the outcomes. Despite the Langendorff method developed over 120 years ago, it has continuously evolved to include new metrics and modes, solidifying its status as the standard for acute preliminary experiments in animal models [42,43]. We also applied a blind, algorithmic protocol for analyzing data. This approach aimed, first of all, to compare all experimental gases and find out whether a specific gas composition is particularly beneficial with a focus on Xe:O_2_ mixtures varied in their gas ratios.

All experimental gases demonstrated superior performance compared to both Control and Air, suggesting that both the Xe additive and its interaction with O_2_ contribute to preservation efficacy. AUC and pressure derivatives correlated closely with HR and LVDP, indicating consistent trends. The estimated infarct area size generally aligned with these results, with control hearts exhibiting the largest infarct areas, experimental gases showing the smallest, and Air group falling in between. To justify potential concerns regarding sample size, we point out that the design of the present study followed a proof-of-concept principle, and thus a small sample size was approved by our local ethical committee (described in methods). We also highlight the asymmetry of statistical inference, meaning the difference is considered established once statistical significance is achieved. Furthermore, a rigorous, standardized data analysis algorithm not only revealed statistically significant differences between the Control and HIPPER groups but also demonstrated the superiority of HIPPER. It was indicated by the large Cohen’s d values, which highlighted the magnitude of these differences. To further address potential reproducibility concerns, we calculated the statistical power of the present study and conducted a second validation experiment using a selected pair (Control vs. Gas B) after the necessary time gap for breeding and raising new rats. Consistent trends were observed in this replication (see Appendix A).

Heart resuscitation results revealed significantly fewer successful launches in the Control group compared to all experimental gases. Two hearts in the Air group also failed to beat. Although a sample size of four animals per group was initially deemed sufficient to demonstrate the HIPPER concept, only one of the four hearts in the Control group was successfully stabilized. Consequently, the number of animals was adjusted to ensure that an adequate number of hearts completed the 1-h reperfusion period, which was essential for assessing cardiac kinetics. Ultimately, only four out of ten hearts in the Control group were resuscitated raising concerns about both two cardioplegias and the six-hour preservation period in HTK. We extensively discuss the limitations of it in corresponding section below. Although potential shortcomings of these limitations exist, exactly similar conditions (including the equal storage time) across all study groups justify the conclusions of this study.

The secondary goal was to identify the optimal gas composition in a blind manner by comparing all metrics simultaneously. Therefore, we intentionally avoided a common trait/character matrix approach and instead deployed PCA. This analysis also yielded consistent results, yet it showed complications upon differentiation between the experimental and air clusters. This could partly be attributed to the well-behaved organs. These organs exhibited noticeable but not distinctly superior benefits across other kinetic variables as well. It can be attributed either to quality of those organs themselves or optimal gas distribution within them. Therefore, methods for measuring gas flow properties may be of special importance. For instance, valves could be calibrated to release excess gas once the desired pressure is reached in the aortic bulb, or other algorithmic approaches can be implemented. Nonetheless, the minimum pressure threshold in organ vasculature has yet to be determined, likely contingent upon organ anatomy. In this study, the pump protocol was tailored to the organ’s requirements and set a priori to operate intermittently (1 s every 2 min), which maintained the pressures in the aortic bulb below 100 mmHg.

Noteworthy, three out of seven hearts in Gas A group were inadequately preserved on occasion. With a 9:1 Xe:O_2_ ratio, Gas A contained sufficient Xe to form gas hydrates (clathrates) within the organ, provided appropriate thermobaric conditions were met [44]. These conditions were easily achieved with a three-bar pressure of the gaseous mixture and storage temperature maintained near 1 °C [Figure S2]. Despite their iced appearance reflecting formation of Xe-water clathrates, these three clathrated organs were able to regain function and beat within one hour upon Langendorff reperfusion, albeit depressive. This was in stark contrast to the lack of rhytm observed in the six hearts preserved using HTK-SCS. Nevertheless, we excluded them from assessment of organ kinetics.

Although gas bubble (embolism) formation is a potential concern persisting among clinicians, a systematic investigation of it during Langendorff reperfusion was beyond the scope of this study. Since it focused on the feasibility of the HIPPER method itself. Nevertheless, we propose at least three mechanisms that might account for their removal: self-dissolution, capillary force, and the Bernoulli effect. Due to the technical complexities involved in such measurements, we considered this a separate research question requiring dedicated hypothesis testing. Future investigations could employ precise gravimetric analysis (comparing heart weights pre- and post-reperfusion) or quantify residual insoluble gas tracers following HIPPER. Regardless of the outcome of such experiments our findings substantiate HIPPER’s therapeutic potential, even if gas bubbles remain.

Further optimization of technical precision—particularly in valve sealing and gas delivery protocols—would improve therapeutic outcomes and enhance the translational relevance of the method. Crucially, such refinements are essential to convince clinicians of its adoptability in real-world practice. The implementation of a disposable sealing circuit and an automated pressure control system, which standardizes compression and decompression steps, would allow for the accurate a priori setting of these parameters and reduce the failure rate. This integrated solution, if designed to be neither complex nor bulky, would present a more compelling alternative to SCS, offering comparable convenience with improved quality.

In conclusion, this study evaluated the efficacy of the HIPPER approach and demonstrated its adaptability to various O_2_ concentrations. A key question arising from our findings pertains to the necessity and optimality of supplemental gases. We incorporated the well-studied Xe gas as an additive, given its established benefits in organ protection [25]. Numerous studies have elucidated its mechanisms at subcellular, biochemical, and molecular levels, confirming its utility in protecting against IRI [32]. While this proof-of-concept study did not delve into the underlying mechanisms, the compelling results suggest that conducting omics studies would be a logical next step to further explore and understand the observed effects. Specifically, spatial transcriptomics could be deployed across various regions of preserved hearts to reveal specific gene expression signatures, followed by gene set enrichment analysis to identify pathways that are significantly over-represented (or underrepresented) in particular preservation methods, whether gaseous or SCS [45]. Similarly, metabolomics studies could provide valuable data on compounds involved in various cell death pathways, helping establish distinct patterns among differently preserved hearts [46]. Additionally, targeted molecular studies would offer insights into specific mechanisms while remaining rapid and cost-effective. In this context, qPCR analysis of ALOX15 or lactylation-related genes (AMPD2, PYGL, SLC7A7, SAT1) might provide preliminary insight on mechanisms of myocardial alteration during storage [46,47].

## 5. Limitations

This study has certain limitations, which we split into three categories: study design, assessment technique (Langendorff) and HIPPER method.

### 5.1. Study Design

The inclusion of pure Xe or pure O_2_ groups would have provided more robust validation of our hypothesis. However, a pure Xe group was deemed unnecessary, as our hypothesis posits that any gas mixture devoid of O_2_ cannot serve as a fuel for HIPPER. Furthermore, we considered a pure O_2_ group redundant due to the well-characterized effects of pure O_2_ in conventional PSF, the closest competitor to HIPPER, as established in prior literature. We deemed the inclusion of two additional groups to be redundant, based on the study’s proof-of-concept design and relevant ethical constraints. We acknowledge that Xe functions primarily as an adjuvant. Therefore, it could theoretically be substituted with less expensive, inert analogs such as argon (Ar) without expecting a direct therapeutic effect. However, this presents a significant challenge, as the entirety of the present work was dedicated to investigating Xe as an adjuvant, specifically its synergistic action with oxygen (O_2_). Consequently, any substitution must be treated as a separate research question, requiring its own experimental design and hypothesis testing.

Another concern is the six-hour duration of storage period. This was selected to mirror the maximum clinically acceptable shelf life for a human heart. However, we suppose this timeframe may not be directly applicable to other species due to differences in basal metabolic rates (BMR) between species. This could explain the significant number of failed hearts in the Control group. Rats have a BMR approximately 6.4 times faster than humans [48]. Thus, this metabolic disparity, combined with the two cardioplegic arrests, may mimic the effects of prolonged storage in rats, accounting for the 60% failure ratio. Indeed, our findings are consistent with the negative trend observed by Galagudza et al. [49], who reported that all five hearts preserved in conventional HTK could not be revived after even two hours of static cold storage, although their study does not explicitly mention the use of two cardioplegic arrests. Conversely, Minasian et al. [50] suggested that less than six hours of static cold storage is insufficient to develop visible signs of infarction, which was a key metric in our study.

### 5.2. Assessment Technique (Langendorff)

The heart resuscitation data presented a challenge for interpretation, as a substantial proportion of hearts in both the Control and Air groups failed to contract after six hours of preservation. We speculate that this outcome may be attributable to the stringent experimental conditions. So, all hearts were subjected to two cardioplegic arrests and a six-hour preservation period. The protocol intentionally included a first cardioplegia to enable safe cannulation via a 21G needle and subsequent accommodation of the heart on the Langendorff rig (as detailed in the Methods). A second cardioplegia was then necessary to reliably arrest the heart after the initial stabilization phase with HTK solution, immediately prior to preservation. This stabilization phase was essential for assessing baseline organ kinetics and compliance with the inclusion criteria. Although this strictness is recommended by experts [28,42,43], we acknowledge that simplifying the protocol—by omitting cannulation, stabilization, or the second cardioplegia, or by reducing storage time—might have increased the resuscitation success ratio.

Another technical limitation is that the least favorable heart’s slices included infarcted papillary muscles, and they also showed non-uniform infarct distribution across all experimental groups. Conversely, these areas were notably absent in the best-performing slices from the Air group and Gases A and B. This discrepancy can be potentially attributed to inadequate left ventricle ballooning, which impeded myocardium drainage through the Thebesian veins. However, this hypothesis requires further confirmation, which was beyond the scope of the present study. Nevertheless, to avoid (or investigate) this confounder future studies in this realm should address variations in isovolumetric contraction, potentially using a more complex ejecting heart Langendorff setup with preload, rather than a balloon approach.

### 5.3. HIPPER Method

The substantial infarct areas in two Air group samples, along with the group’s overall functional variation, may stem from the HIPPER method’s sensitivity. Improper aortic cannulation—potentially causing insufficient valve closure and flawed gas flow—highlights how critical precise organ accommodation and connection are for consistent results. This concern was extensively discussed by Fischer, advocating for an aortic valve guard to prevent gas efflux into left ventricle during coronary PSF in porcine hearts [20,51]. However, tailoring aortic valves for small rat hearts to prevent gas leakage from the coronary vasculature was unfeasible. Instead, we minimized the 21G cannula length while kept sufficient aortic length to avoid occluding the coronary orifices. Nevertheless, we acknowledge potential confounding factors, such as aortic valve insufficiency or anatomical variations in ligament structures, which might have affected coronary gas flow. To account for this, we compared cardiac kinetic parameters in significantly infarcted hearts against the group mean. Although all kinetic measurements fell within one or two standard deviations, the worst-performing hearts exhibited notably impaired metrics, suggesting a plausible relationship between infarct severity, cardiac kinetics, and suboptimal cannulation.

## 6. Conclusions

Our results further indicated that HIPPER effectively delivered the Xe:O_2_ mixture to the organ, consistent with the design of our experimental rig. Of crucial importance, this mixture was capable of solely fueling the organ throughout preservation without any fluidic additives, as evidenced by both qualitative and quantitative data. Likewise, the HIPPER method, when supplemented with gases at various Xe:O_2_ ratios, outperformed traditional SCS in HTK solution for preserving rat hearts over a six-hour period. Moreover, HIPPER using Air either matched or surpassed the conventional technique in efficacy. Within the scope of this study, no dependency on Xe concentration was observed, and a stable effect was demonstrated within the effective range. However, high Xe content might cause undesirable clathrate formation which is harmful. Therefore, using mixtures with less Xe:O_2_ ratio are beneficial especially since they are cheaper. These findings underscore the translational potential of HIPPER as closed robust alternative to PSF and highlight the need for its validation in larger animal models as a critical next step.

## 7. Patents

The above-described HIPPER technology was developed by InPres LLC and is protected by a patent RU2024102293 and the international application WO2025165261: “Donor organ preservation method and donor organ preservation container”.

## Data Availability

All raw data related to this study is deposited in an external repository BioStudies (accession number S-BSST2120). The deposited archive includes detailed descriptions for navigating the raw data files as well as .txt files for two independent experiments. R scripts for data processing are available upon request.

## Supplementary Materials

The following supporting information can be downloaded at: https://www.mdpi.com/article/10.3390/pathophysiology32040058/s1, Table S1: Input measures of animals and organs, Table S2: PCA output processed by PerMANOVA, Table S3: Including criteria for hearts, Figure S1: Clathrated heart with data from sensors, File S1: GIF movie of HIPPER vs. Control.

## Abbreviations

The following abbreviations are used in this manuscript:

WIT	warm ischemia time
DCD	donation after circulatory death
DBD	donation after brain death
NMP	normothermic machine perfusion
SCS	static cold storage
SNMP	Sub-normothermic machine perfusion
HMP	hypothermic machine perfusion
PSF	persufflation
HTK	histidine tryptophan ketoglutarate
TTC	Triphenyltetrazolium chloride
HIPPER	High-Pressure Perfusion technique
Dp/dt	derivative pressure of time
AUC	area under curve
ANOVA	analysis of variance
perMANOVA	permutation ANOVA
PCA	Principal component analysis
IRI	ischemia–reperfusion injury
HR	heart rate
CFR	coronary flow rate
LVDP	left ventricle developed pressure

## Appendix A

The statistical power of the present study was calculated for ANOVA followed by Student’s pairwise comparisons between two crucial variables (HR and LVDP) reflecting overall heart viability. The power was 0.996 for HR and 0.97 for LVDP in the Control vs. Gas B comparison, selected as most cost-effective for routine usage given the lack of differences across all gaseous mixtures in PerMANOVA. We also calculated power for dichotomous heart launch data, showing values of 0.85 and 0.99 for separated and pooled gaseous groups, respectively, as reflected in Figure 5. All these statistical power outputs suggest that with the above estimated probabilities, the same significant differences could be detected in another independent experiment, as these probabilities appear quite high. Indeed, we observed the same trend in significance when executing the second experiment, which revealed exactly similar pairwise differences in both merged and separate data analysis. Nonetheless, we intentionally did not provide the reader by merged the data, considering both experiments as independent. This study presents data from the original experiment only, but for those interested in independent analysis, we provide deposited data from both experiments separately.
