# Supplementary material for: Improved Rat Heart Preservation Using High-Pressure Gaseous Perfusion with Oxygen–Xenon Mixture
Source: Pathophysiology. 2025 Oct 31;32(4):58. doi: 10.3390/pathophysiology32040058 (PMC12642012; doi:10.3390/pathophysiology32040058)
Supplement: Supplementary file 1 [file pathophysiology-32-00058-s001.zip › pathophysiology-3954493-supplementary/Figure S1. Clathrated heart with data from sensors.docx]

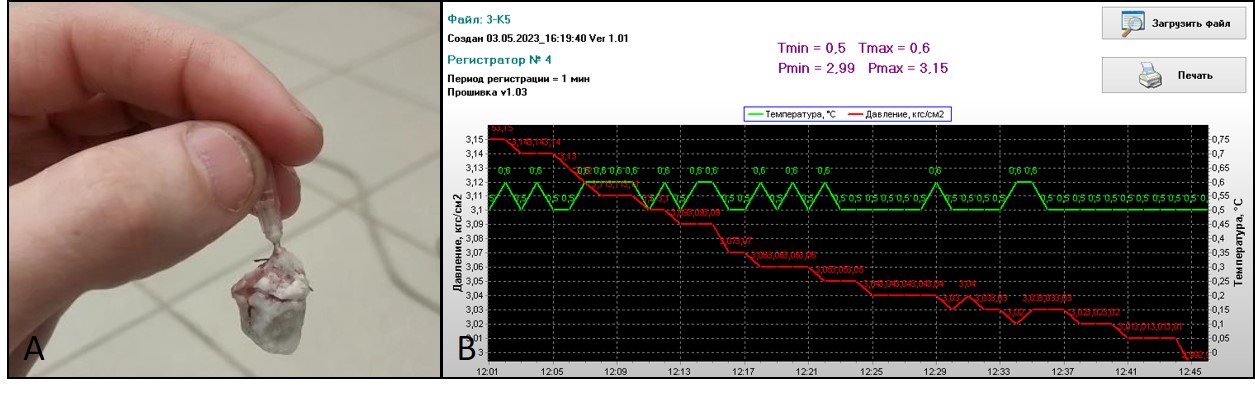


Legend (B):

- Left y-axis denotes pressure inside sealed chamber in bars (red line)
- Right y-axis denotes temperature in ^0^C (green line)
- X-axis denotes time (4-min step)

Fig. S1. (A) Picture of heart underwent unintended clathrate formation upon HIPPER storage (over 3 bars) in Gas A. (B) 45-min fragment of temperature-pressure data of above (clathrated) experiment. Steep red curve indicates onset of excessed gas dissolution accompanying clathrate formation accompanied by substantial pressure decline in inner chamber. Green line points out on flat temperature curve, just with 0.1 ^0^C temperature cycling hereby better mimics SCS environment.
