# Supplementary material for: Improved Rat Heart Preservation Using High-Pressure Gaseous Perfusion with Oxygen–Xenon Mixture
Source: Pathophysiology. 2025 Oct 31;32(4):58. doi: 10.3390/pathophysiology32040058 (PMC12642012; doi:10.3390/pathophysiology32040058)
Supplement: Supplementary file 1 [file pathophysiology-32-00058-s001.zip › pathophysiology-3954493-supplementary/File S1. GIF movie of HIPPER vs Control.docx]

File. S1. GIF pictures show results of preliminary in vivo pilot study (n=4) aimed to estimate the principal feasibility of HIPPER technique as measured by presence of sustained rhythm upon heterotopic (to abdominal area) transplantation. !NB: Software to open .ppt (e.g. PowerPoint) files must be installed prior to double-click on image to browse
