# Supplementary material for: Improved Rat Heart Preservation Using High-Pressure Gaseous Perfusion with Oxygen–Xenon Mixture
Source: Pathophysiology. 2025 Oct 31;32(4):58. doi: 10.3390/pathophysiology32040058 (PMC12642012; doi:10.3390/pathophysiology32040058)
Supplement: Supplementary file 1 [file pathophysiology-32-00058-s001.zip › pathophysiology-3954493-supplementary/Table S1. Input measures of animals and organs.docx]

Table S1. Initial parameters of animals included in the study.

| **Group** | **Heart rate, beats per minute** | **Coronary flow rate, ml/min** | **Left ventricular pressure, mmHg** | **dP/dT max, mmHg/sec** | **dP/dT min, mmHg/sec** | **Langendorff stabilization time, sec** | **Cardioplegic flow time, sec** | **Animal’s age, days** | **Animal’s weight, g** |
| --- | --- | --- | --- | --- | --- | --- | --- | --- | --- |
| Control (HTK solution) | 256±18 | 15.6±3.5 | 123±9 | 2418±266 | -1894±177 | 614±26 | 223±25 | 114±24 | 346±32 |
| Air | 260±21 | 14.7±1.4 | 124±12 | 2418±258 | -1997±208 | 626±16 | 211±18 | 108±9 | 348±31 |
| Gas A | 260±25 | 13.2±1.1 | 125±10 | 2484±338 | -1875±274 | 627±22 | 230±23 | 120±33 | 343±20 |
| Gas B | 242±28 | 13.7±1.7 | 134±8 | 2627±233 | -1980±127 | 620±31 | 245±15 | 107±10 | 351±17 |
| Gas C | 263±8 | 16.3±1.7 | 121±3 | 2330±54 | -1882±60 | 635±34 | 249±14 | 106±8 | 369±15 |

Values indicated as mean±SD. There was no difference (p>0.05) upon group comparison in all variables
