# Supplementary material for: Improved Rat Heart Preservation Using High-Pressure Gaseous Perfusion with Oxygen–Xenon Mixture
Source: Pathophysiology. 2025 Oct 31;32(4):58. doi: 10.3390/pathophysiology32040058 (PMC12642012; doi:10.3390/pathophysiology32040058)
Supplement: Supplementary file 1 [file pathophysiology-32-00058-s001.zip › pathophysiology-3954493-supplementary/Table S2. PCA output processed by PerMANOVA.docx]

Table S2. Permutational ANOVA (PerMANOVA) output of comparison of Cartesian distances between groups represented by PCA.

| **Pairs** | **Degrees of freedom** | **SumsOfSqs** | **F.Model** | **R^2** | **p.value** |
| --- | --- | --- | --- | --- | --- |
| Air vs Control | 1 | 0,1210876 | 2,05260986 | 0,20418676 | 0,19 |
| Air vs Gas C | 1 | 0,25846544 | 4,12517175 | 0,34021553 | 0,1 |
| Air vs Gas A | 1 | 0,21036076 | 2,99481225 | 0,27238412 | 0,084 |
| Air vs Gas B | 1 | 0,23398509 | 3,72437241 | 0,31766071 | 0,13 |
| Control vs Gas C | 1 | 0,57189983 | 43,2564142 | 0,87818845 | 0,032 |
| Control vs Gas A | 1 | 0,50278902 | 21,545754 | 0,78218059 | 0,039 |
| Control vs Gas B | 1 | 0,52512031 | 39,0499067 | 0,86681437 | 0,03 |
| Gas C vs Gas A | 1 | 0,00684217 | 0,24245169 | 0,03883918 | 0,913 |
| Gas C vs Gas B | 1 | 0,00550526 | 0,30030379 | 0,04766497 | 0,809 |
| Gas A vs Gas B | 1 | 0,00484863 | 0,1704444 | 0,02762271 | 0,809 |
|  |  |  |  |  |  |

Legend

- Pairs: pairwise comparisons
- Degrees of freedom, SumOFSqs, F.Model and R^2: output parameters of the PerMANOVA model
- p.value: significance level (probability of α)
- Pairwise significant differences (p<0,05) colored in red
- Likely pairwise trends (0,05<p<0,1) colored in green
