# Supplementary material for: Improved Rat Heart Preservation Using High-Pressure Gaseous Perfusion with Oxygen–Xenon Mixture
Source: Pathophysiology. 2025 Oct 31;32(4):58. doi: 10.3390/pathophysiology32040058 (PMC12642012; doi:10.3390/pathophysiology32040058)
Supplement: Supplementary file 1 [file pathophysiology-32-00058-s001.zip › pathophysiology-3954493-supplementary/Table S3. Including criteria for hearts.docx]

Table S4. Heart inclusion criteria applied across all experiment

| **Variable** | **Value** |
| --- | --- |
| Time to perfusion | <3 min |
| Coronary flow rate | 8 - 28 ml/min |
| Arrhythmia duration | <3 min |
| Heart rate | 120 - 400 beats/min |
| Left ventricular pressure | 90 - 150 mmHg |
